# Determining risk features for medulloblastoma in the molecular era

**DOI:** 10.1093/neuonc/noae223

**Published:** 2024-10-25

**Authors:** Nicholas G Gottardo, Amar Gajjar

**Affiliations:** Department of Pediatric and Adolescent Oncology and Hematology, Perth Children’s Hospital, Perth, Western Australia, Australia; Brain Tumor Research Program, Kids Cancer Centre, The Kids Research Institute Australia, University of Western Australia, Perth, Western Australia, Australia; Department of Pediatric Medicine, St. Jude Children’s Research Hospital, Memphis, Tennessee, USA

Since the discovery of medulloblastoma being a compendium of 4 individual molecular diseases, each with a distinct cell of origin, molecular and clinical features as well as outcome, investigators have attempted to enhance the risk stratification of these tumors to refine therapy.^1^ Radiation therapy remains the most effective therapy for medulloblastoma, with attempts to either avoid or reduce the volume of radiation therapy for patients even with the most favorable subgroup, Wingless medulloblastoma, resulted in early disease progression.^2^ Accurate risk stratification facilitates safe reduction in radiation therapy dose, which is the main adverse factor impacting neurocognitive and neuroendocrine outcomes.

Methylation profiling has further refined Sonic Hedgehog (SHH) medulloblastoma into 4 subtypes and Group 3 and Group 4 medulloblastoma into 8 subtypes. Using advanced genomic tools several investigators have defined risk features in their patient cohorts thus providing a robust risk classification based on several prospectively treated patient cohorts.^3,4^ While the specific therapy in each of the patient series differs, the essential treatment components consist of maximal safe surgical resection, cranio-spinal irradiation (CSI), and chemotherapy (whose intensity and timing differ).

In this issue of *Neuro-oncology,* Massimino et al.^5^ report the long-term outcomes of their Milano-hyperfractionated accelerated radiotherapy (HART) strategy for patients > 3 years old with high-risk medulloblastoma. The strategy involved multiagent high-dose chemotherapy cycles, with adjusted HART based on age and response, followed by additional cycles of thiotepa-based chemotherapy with autologous stem cell rescue. Their original series which included 33 patients with metastatic disease, reported a 5-year event-free survival (EFS) of 70% ± 8%, which compared favorably with other approaches.^6^ This update describes a total of 89 patients, with the eligibility criteria modified to include large cell/anaplastic histological subtypes, TP53 mutations, and/or *MYC* and *MYCN* amplification to the original clinical criteria. A detailed retrospective analysis of 66/89 patients using methylation profiling further revealed the molecular group and biological risk features. The details of the disease characteristics are presented in their manuscript. This report revealed a 5-year EFS of 68.2%, confirming the originally documented survival rate, and the authors conclude that response to neoadjuvant chemotherapy was the main determinant of survival in this updated series. Other features, such as the presence of metastatic disease, *MYC/MYCN* amplification, post-surgical residual disease, histological subtype, CSI dose reduction (31.2 Gy instead of 39 Gy), and omission of radiation therapy boosts, did not significantly impact survival. These findings are provocative and contrary to risk features that have been previously documented in much larger cohorts,^1,7,8^ including 3 molecularly annotated prospective clinical trials^3,4,9^ (summarised in Table 1). Despite the long study duration of 20 years, the results must be interpreted with caution due to the relatively small number of patients included in the study. Additionally, the therapy used in this study is different from what is used in most other published series, which may have impacted the results.

**Table 1.** Survival Outcomes for High-Risk Medulloblastoma Patients With Molecular Analyses Treated on Clinical Trials With Comparison to the Milano-HART Strategy and Associated Prognostic Significance of *MYC/N* Amplification and Metastatic Disease. Also Shown is Data From Multiple Large Retrospective Cohorts of Medulloblastoma Patients

Study	Type of study	*n* patients	5-year EFS (95% CI) HR MB patients	5-year OS (95% CI) HR MB patients	Prognostic significance of *MYC* amp	Prognostic significance of *MYCN* amp	Prognostic significance of M+ disease
Milano-HART strategy^5^	Retrospective analysis of uniformly treated series at a single institution	89	68.2%	75.9%	Not prognostic	Not prognostic	Not prognostic
St Jude SJMB03^3^ (only HR MB patients)	Prospective clinical trial	103	56.7% (SE 4.9%)	69.5% (SE 4.6%)	Inferior survival in Group 3	Inferior survival in SHH	Inferior prognosis in SHH, Group 3 and Group 4
COG 0332^4^	Prospective clinical trial	261	62.9% (55.6–70.2%)	73.4% (66.7–80.1%)	Inferior survival in Group 3	N/R	Inferior prognosis in Group 3
HIT-SKK 2000^9^	Prospective clinical trial	123	62% (52–72%)	74% (66–82%)	^*^Inferior survival (esp. Group 3)	^*^Inferior survival	N/A only M+ patients included
^#^International meta-analysis^1^	Meta-analysis of 7 studies (retrospective)	550 (additional 402 patients in a validation cohort)	N/A	N/A	Inferior OS in multivariate analysis	Inferior OS in multivariate analysis	Inferior OS
^@^MAGIC & Int.CC^7^	Retrospective analysis of patients from multiple global centers	1126 (673 in the discovery cohort) (453 in the validation cohort)	N/A	N/A	Inferior OS in Group 3	Inferior OS in SHH	Inferior prognosis in SHH, Group 3 and Group 4 (without Chr11 loss or Chr17 gain)
^$^European consortium^8^	Retrospective analysis of patients from multiple centers/studies in Europe	704 (428 in the discovery cohort) (276 in the validation cohort)	N/A	N/A	Independent negative prognostic marker in Groups 3/4 combined	Independent negative prognostic marker in the SHH group	Independent negative prognostic marker in SHH group. Negative prognostic marker by univariate analysis in Groups 3/4 combined

Amp, amplification; Chr, chromosome; CCLG, Children’s Cancer and Leukaemia Group; CI, confidence interval; COG, Children’s Oncology Group; EFS, event free survival; UKCCSG-SIOP-PNET3, United Kingdom Children’s Cancer Study Group SIOP-PNET3 clinical trial; HR MB, high-risk medulloblastoma; Int.CC, International Collaborating Centers; MAGIC, Medulloblastoma Advanced Genomics International Consortium; OS, overall survival; N/A, not applicable; N/R, not reported; SE, standard error, SHH, Sonic Hedgehog group.

^*^ *MYC/MYCN* were combined for survival analysis.

^$^European consortium consisting of UK CCLG (Children’s Cancer and Leukaemia Group), European institutions, and UKCCSG-SIOP-PNET3; included only children 0 to 16 years (with both average and high-risk medulloblastoma).

^@^Study included infants, children (with both average and high-risk medulloblastoma), and adults.

^#^Study included infants, children (with both average and high-risk medulloblastoma), and adults.

Risk features are an interplay between therapy given to a cohort of patients and the molecular features of the tumors in the cohort. The methodology used to determine the molecular features of the tumor also impacts the accuracy of the results. Technologies that are currently being used to determine an integrated diagnosis as recommended by the latest version of the World Health Organization (WHO) guidelines include histopathological examination (tumor morphology) methylation profiling (group and chromosomal copy number changes); sequencing of the tumor (whole exome or panel sequencing); and germline sequencing (to determine cancer predisposition syndromes). Indeed, in North America, radiotherapy is delivered upfront, followed by a different, albeit alkylator-based, maintenance chemotherapy regimen. In these studies, the rate of progressive disease during radiotherapy is very low, negating a similar response-based assessment. Integrated diagnosis on a robust prospective trial cohort of patients is most likely to yield risk features that can be interpreted in the context of a given therapy. Consequently, caution should be exercised before the clinical implementation of a modified risk stratification. Importantly, to this point, the Milano-HART treatment strategy is currently being prospectively tested in one of the arms of the ongoing International Society for Paediatric Oncology-Europe (SIOP-E) high-risk medulloblastoma clinical trial (SIOP-HR-MB),^10^ which will definitively confirm or refute their findings.

In summary, the evolution of risk features for medulloblastoma is inevitable as treatment modalities change and therapies become further refined. The current report and several other recently published prospective clinical trials, which included extensive molecular analyses, provide opportunities for improved risk classification and treatment refinement. The subsequent series of medulloblastoma trials will incorporate molecular data at diagnosis^3^ and the inclusion of serial CSF analysis for the detection of minimal residual disease^11^ and provide opportunities for either therapy augmentation or de-escalation.

## References

[1] Kool M , Korshunov A, Remke M, et al Molecular subgroups of medulloblastoma: An international meta-analysis of transcriptome, genetic aberrations, and clinical data of WNT, SHH, Group 3, and Group 4 medulloblastomas. Acta Neuropathol. 2012;123(4):473–484. 22358457 10.1007/s00401-012-0958-8 PMC3306778

[2] Gottardo NG , Gajjar A. Verschlimmbesserung: Craniospinal radiotherapy is essential in WNT medulloblastoma patients. Clin Cancer Res. 2023;29(24):4996–4998. 37823794 10.1158/1078-0432.CCR-23-2331 PMC10722133

[3] Gajjar A , Robinson GW, Smith KS, et al Outcomes by clinical and molecular features in children with medulloblastoma treated with risk-adapted therapy: Results of an International Phase III Trial (SJMB03). J Clin Oncol. 2021;39(7):822–835. 33405951 10.1200/JCO.20.01372 PMC10166353

[4] Leary SES , Packer RJ, Li Y, et al Efficacy of carboplatin and isotretinoin in children with high-risk medulloblastoma: A randomized clinical trial from the Children’s Oncology Group. JAMA Oncol 2021;7(9):1313–1321. 34292305 10.1001/jamaoncol.2021.2224 PMC8299367

[5] Massimino M , Barretta F, Dossena C, et al Long-term outcome of the Milano-HART strategy for high-risk medulloblastoma, including the impact of molecular subtype. Neuro Oncol. 2024;27(1):209–219. 10.1093/neuonc/noae189 PMC11726337 39331528

[6] Gandola L , Massimino M, Cefalo G, et al Hyperfractionated accelerated radiotherapy in the Milan strategy for metastatic medulloblastoma. J Clin Oncol. 2009;27(4):566–571. 19075266 10.1200/JCO.2008.18.4176

[7] Shih DJ , Northcott PA, Remke M, et al Cytogenetic prognostication within medulloblastoma subgroups. J Clin Oncol. 2014;32(9):886–896. 24493713 10.1200/JCO.2013.50.9539 PMC3948094

[8] Schwalbe EC , Lindsey JC, Nakjang S, et al Novel molecular subgroups for clinical classification and outcome prediction in childhood medulloblastoma: A cohort study. Lancet Oncol. 2017;18(7):958–971. 28545823 10.1016/S1470-2045(17)30243-7 PMC5489698

[9] von Bueren AO , Kortmann RD, von Hoff K, et al Treatment of children and adolescents with metastatic medulloblastoma and prognostic relevance of clinical and biologic parameters. J Clin Oncol. 2016;34(34):4151–4160. 27863192 10.1200/JCO.2016.67.2428

[10] Bailey S , André N, Gandola L, et al Clinical trials in high-risk medulloblastoma: Evolution of the SIOP-Europe HR-MB trial. Cancers (Basel). 2022;14(2):374. 35053536 10.3390/cancers14020374 PMC8773789

[11] Liu APY , Smith KS, Kumar R, et al Serial assessment of measurable residual disease in medulloblastoma liquid biopsies. Cancer Cell. 2021;39(11):1519–1530.e4. 34678152 10.1016/j.ccell.2021.09.012 PMC9620970

